# KSR2 functions as a metabolic checkpoint for anti-PD-1 resistance by reprogramming glucose metabolism

**DOI:** 10.1007/s00262-026-04394-z

**Published:** 2026-04-21

**Authors:** Yuli Ge, Qiong Zhou, Qiangqiang Zhang, Yanyan Hu, Rui Wang

**Affiliations:** 1 Department of Medical Oncology, Jinling Clinical Medical College, Nanjing University of Chinese Medicine, Nanjing, Jiangsu Province China; 2 Department of Medical Oncology, Nanjing Jinling Hospital, Affiliated Hospital of Medical School, Nanjing University, Nanjing, Jiangsu Province China; 3 Department of Medical Oncology, Jinling Hospital, Nanjing Medical University, Nanjing, Jiangsu Province China

**Keywords:** Anti-PD-1, Drug resistance, Glycolysis, Metabolic reprogramming, KSR2

## Abstract

Immune checkpoint blockade targeting the PD-1/PD-L1 axis has revolutionized cancer therapy, yet the frequent emergence of resistance limits its clinical efficacy. Elucidating the mechanisms underlying resistance and developing effective strategies remain critical challenges in tumor immunotherapy. This study identifies kinase suppressor of Ras 2 (KSR2) as a driver of resistance to anti-PD-1 therapy in lung cancer. Transcriptomic analysis of an anti-PD-1-resistant mouse model and public clinical datasets revealed upregulation of KSR2 in resistant tumors. In vivo functional studies demonstrated that KSR2 overexpression is sufficient to confer resistance, while its knockdown resensitizes tumors to PD-1 blockade. Mechanistically, KSR2 functions as a central metabolic checkpoint, driving profound glucose metabolic reprogramming in cancer cells by enhancing glucose uptake, potentiating the Warburg effect, promoting lactate accumulation, and disrupting the tricarboxylic acid cycle. This metabolic reprogramming was subsequently associated with an immunosuppressive tumor microenvironment, characterized by reduced infiltration and impaired function of CD8⁺ T cells, alongside an enrichment of regulatory T cells. These findings suggest that KSR2 plays a role in modulating immunotherapy response, indicating a potential link between tumor metabolism and immune evasion. KSR2 emerges as a candidate target for further exploration in overcoming anti-PD-1 resistance.

**Supplementary Information:**

The online version contains supplementary material available at 10.1007/s00262-026-04394-z.

## Introduction

Lung cancer remains the leading cause of cancer-related mortality worldwide, with non-small cell lung cancer (NSCLC) accounting for approximately 85% of cases [1–3]. The treatment landscape for advanced NSCLC has been reshaped by immune checkpoint blockade (ICB) targeting the programmed cell death protein 1 (PD-1) pathway [4, 5]. Despite remarkable clinical successes, intrinsic and adaptive resistance limits durable responses to anti-PD-1 therapy, leaving the majority of patients with limited therapeutic options. Overcoming this resistance constitutes a central challenge in oncology.

Mechanisms of immune escape are multifaceted, encompassing aberrant tumor cell signaling, impaired antigen presentation, T cell exhaustion, and the establishment of an immunosuppressive tumor microenvironment (TME) [6–8]. Recently, tumor metabolic reprogramming has emerged as a critical facilitator of immune evasion [9–11]. Compelling evidence indicates that heightened glycolytic flux in cancer cells can acidify the TME and deprive infiltrating immune cells of critical nutrients, thereby blunting anti-tumor immunity and diminishing the efficacy of ICB [12, 13]. However, the key upstream regulators that orchestrate this metabolic immunosuppression remain poorly defined.

Kinase suppressor of Ras 2 (KSR2), a scaffolding protein well known for its role in energy homeostasis and metabolic regulation [14], represents a compelling candidate bridging oncogenic signaling with metabolic rewiring. Nevertheless, its potential function in modulating the immune TME and response to immunotherapy is entirely unexplored.

Through transcriptomic analysis of a novel anti-PD-1-resistant mouse model and clinical datasets, we identify KSR2 as a key mediator of immunotherapy resistance. Using lentiviral-mediated gain- and loss-of-function studies in vivo, we demonstrate that KSR2 is both necessary and sufficient for driving anti-PD-1 resistance. Integrated analyses combining metabolomics with immune profiling, including flow cytometry and multiplex immunofluorescence, reveal that KSR2-driven metabolic reprogramming is associated with an immunosuppressive tumor microenvironment. This metabolic reprogramming, characterized by enhanced glucose uptake, augmented aerobic glycolysis, and disruption of the tricarboxylic acid cycle, correlates with reduced infiltration and impaired function of CD8⁺ T cells, alongside an enrichment of regulatory T cells.

We therefore hypothesize that KSR2 functions as a metabolic checkpoint linking oncogenic processes to immune modulation by reprogramming tumor glucose metabolism. This study aims to investigate the role and underlying mechanisms of KSR2 in anti-PD-1 resistance, which may provide a novel therapeutic target and conceptual framework for overcoming anti-PD-1 resistance in NSCLC.

## Material and methods

### Cell lines and culture

The murine Lewis lung carcinoma (LLC) cell line was obtained from the Cell Bank of the Chinese Academy of Sciences. LLC cells were cultured in high-glucose DMEM medium (C11995500BT, Gibco) supplemented with 10% fetal bovine serum (FSP500, ExCell) and 1% penicillin–streptomycin (C100C5, NCM). All cells were maintained at 37°C in a humidified incubator with 5% CO₂.

### Animal studies and establishment of resistance models

#### Animals and anti-PD-1-resistant models

Female C57BL/6J mice (6 weeks old) were obtained from Nanjing Huimiaoxin Biotechnology Co., Ltd. (Nanjing, China) and maintained under specific pathogen-free conditions.

To generate anti-PD-1-resistant models, LLC cells (2 × 10⁶cells/100*μ*l PBS) in logarithmic growth phase were subcutaneously injected into the right flank of C57BL/6J mice. Seven days post-inoculation, mice were randomized into two groups and treated with either IgG (BE0089, Bio X Cell) or anti-mouse PD-1 antibody (BE0146, Bio X Cell) at 200*μ*g per dose, administered every 3 days. Tumor volume was measured every 3 days and calculated as *V* = ½ab^2^. After four treatment cycles, tumors were harvested and transplanted into new mice. This selection process was repeated four times until tumor growth under anti-PD-1 treatment matched that of the IgG group, indicating established resistance. Primary cells from resistant tumors were passaged to establish the LLC-R cell line.

#### Animal experiment

LLC, LLC-R, *Ksr2*-OENC, *Ksr2*-OE, *Ksr2*-shNC, and *Ksr2*-sh cells were inoculated into C57BL/6J mice. Seven days later, mice received either anti-PD-1 antibody or IgG (200*μ*g, every 3 days). Tumor size was recorded every 3 days, and survival was monitored throughout the study.

### RNA sequencing (RNA-seq) analysis

Transcriptomic profiling of tumor tissues was performed on the Illumina platform (Beijing Novogene Biotech Co., Ltd. Beijing, China). We identified differentially expressed genes (DEGs) (padj < 0.05 and |log2FC|> 1) and performed subsequent enrichment analyses for GO, KEGG, and Reactome pathways.

### RNA extraction and reverse transcription quantitative PCR (RT-qPCR)

Total RNA was extracted using RNAex Pro Reagent (AG21101, Accurate Biology, China) and reverse-transcribed into cDNA with the Evo M-MLV RT Master Mix (AG11706, Accurate Biology) according to the manufacturer’s instructions. qPCR was then performed using the SYBR Green Pro Taq HS Premix (AG11718, Accurate Biology) on a QuantStudio 1 system (Thermo Fisher Scientific). The thermal cycling conditions were as follows: initial denaturation at 95°C for 30 s; 40 cycles of 95°C for 5 s and 60°C for 30 s. Primer sequences are listed in TABLE S1.

### Western blotting (WB)

Proteins were extracted with RIPA lysis buffer (P0013B, Beyotime) containing protease and phosphatase inhibitor cocktail (P002, NCM). Protein concentration was determined using a BCA protein assay kit (ZJ102, Epizyme Biotech). Protein lysates were separated by SDS-PAGE, transferred to PVDF membranes, and probed with primary antibodies against KSR2 (CSB-PA581207, CUSABIO) and GAPDH (10,494–1-AP, Proteintech). Blots were then incubated with an HRP-conjugated secondary antibody, and signals were detected by enhanced chemiluminescence.

### Cell transduction and generation of stable cell lines

Stable Ksr2-overexpressing LLC cells were generated by lentiviral transduction using Lenti-*Ksr2*-OE, with Lenti-NC (both from GeneChem, China) as a control. For stable knockdown, LLC-R cells were transduced with lentiviral particles expressing *Ksr2*-specific shRNA or a non-targeting control (GeneChem, China). Transduction was performed in the presence of HitransG A enhancer. Cells were selected with 2*μ*g/mL puromycin for 6–8 days. Efficiency was monitored by GFP fluorescence, and KSR2 expression was validated by RT-qPCR and Western blotting. The primers for lentiviral vector construction are listed in TABLE S2.

### Flow cytometry analysis

Tumors were dissociated into single-cell suspensions using a tumor dissociation kit (130–096-730, Miltenyi Biotec). After red blood cell lysis (ZYFB006-0500, ZUNYAN), viable cells were stained with FVD-APCCY7 (65–0865-14, eBioscience). Surface staining was performed with antibodies against CD45-PE-Cy5.5 (35–0451-82, eBioscience), CD3-FITC (11–0032-82, eBioscience), CD4-EF450 (48–0041-82, eBioscience), CD8-EF506 (69–0081-82, eBioscience), and CD25-APC (17–0251-82, eBioscience). Cells were then fixed and permeabilized (Foxp3/Transcription Factor Staining Buffer Set, 00–5523-00, eBioscience) and stained intracellularly with anti-FoxP3-PE (12–5773-82, eBioscience).

The gating strategy was as follows (Fig. S3): Live cells were identified using a viability dye. Single cells were gated based on forward scatter area versus height (FSC-A vs. FSC-H). From these, CD45⁺ leukocytes were selected. Subsequently, CD3⁺ T cells were gated from the CD45⁺ population. CD4⁺ and CD8⁺ T cell subsets were then analyzed as proportions of CD3⁺ T cells. Regulatory T cells were specifically defined as CD4⁺CD25⁺FoxP3⁺ cells, and their frequency is reported as a percentage of CD4⁺ T cells.

### Enzyme-linked immunosorbent assay (ELISA)

Mouse tumor homogenates were prepared, and concentrations of granzyme B and IFN-γ were measured using the mouse granzyme B ELISA kit (E-EL-M0594, Elabscience) and mouse IFN-γ ELISA kit (E-HSEL-M0007, Elabscience), respectively, according to the manufacturers’ protocols.

### *Immunohistochemistry (IHC) and (*multiplex) immunofluorescence (MIF/IF)

Tumor samples were fixed in 4% paraformaldehyde, embedded in paraffin, and sectioned. After deparaffinization, antigen retrieval, and blocking, IHC staining involved primary and HRP-conjugated secondary antibodies, with signals visualized by DAB. IF and multiplex IF were carried out employing a TSA-based method, which comprised sequential cycles of primary antibody, HRP secondary, and fluorophore tyramide, interrupted by heat-mediated stripping. Prior to imaging, nuclei were counterstained with DAPI.

Ksr2 (1:200, CSB-PA581207, CUSABIO); LDHA (1:300, 19,987–1-AP, Proteintech); HK2 (1:4000, 66,974–1-Ig, Proteintech); HIF-1α (1:100, ab179483, Abcam); CD8 (1:1000, ab217344, Abcam), granzyme B (1:800, 13,588–1-AP, Proteintech), FOXP3 (1:1000, GB112325, Servicebio); HRP-polymer anti-rabbit IHC kit (KIT-5005, Fuzhou Maixin Biotech) were used.

### *Targeted metabolomics analysis of* central energy metabolism *intermediates*

Targeted metabolomics profiling of central energy metabolism pathways was performed by Metabo-Profile Biotechnology Co., Ltd. (Shanghai, China). Briefly, metabolites were extracted from snap-frozen tumor tissues or cell pellets using a methanol/water system. The analysis specifically targeted intermediates of glycolysis, the pentose phosphate pathway (PPP), the tricarboxylic acid (TCA) cycle, and related organic acids. Quantification was achieved using ultra-performance liquid chromatography coupled with tandem mass spectrometry (UPLC-MS/MS). Differential metabolites were identified by t test or Mann–Whitney U test, and one-way ANOVA. The pathway enrichment analysis was performed using SMPDB and Predicted Metabolite Sets databases.

## Results

### Successful establishment of an anti-PD-1-resistant mouse model

**Fig. 1:**
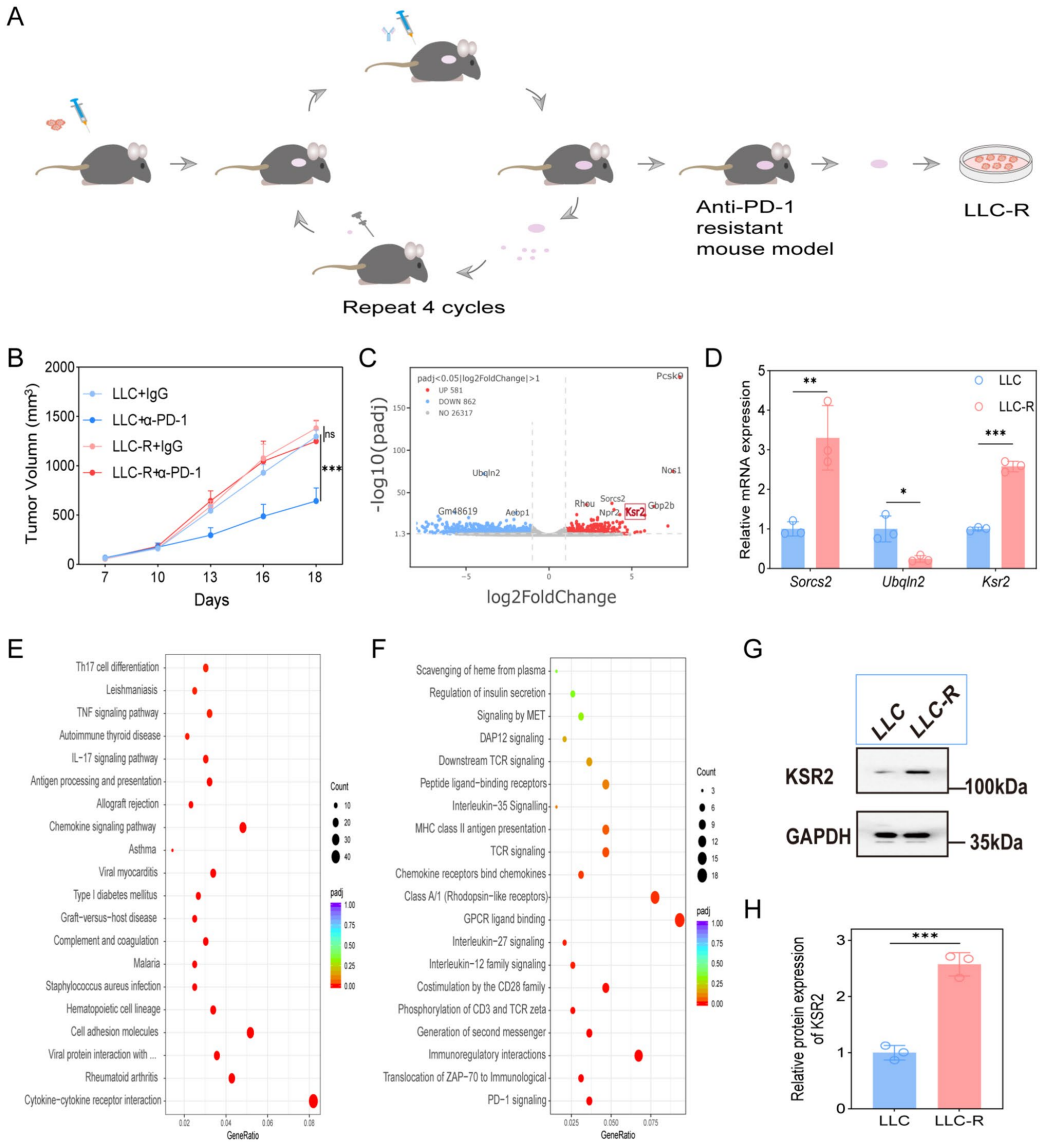
KSR2 is upregulated in anti-PD-1-resistant mouse model. **A** Establishment of the anti-PD-1-resistant mouse model and the resistant cell line (LLC-R). **B** Tumor growth curves of mice bearing parental LLC or LLC-R tumors treated with anti-PD-1 or IgG control (*n* = 6 mice). Two-way ANOVA***P < 0.001; ns, not significant. **C** Volcano plot of DEGs from RNA-seq analysis of LLC versus LLC-R tumors. **D** RT-qPCR validation of selected DEGs in LLC and LLC-R cells. Mean ± SD (*n* = 3 independent experiments), unpaired two-tailed t test *P < 0.05, **P < 0.01, ***P < 0.001. **E**, **F** Pathway enrichment analysis of DEGs using KEGG (**E**) and Reactome (**F**) databases. **G** Representative Western blots of protein KSR2 and GAPDH in LLC and LLC-R cells. Blots are representative of three independent experiments. **H** Densitometric quantification of protein KSR2 levels normalized to GAPDH. Mean ± SD (*n* = 3 independent experiments), unpaired two-tailed t test, ***P < 0.001

To investigate the underlying mechanisms of acquired resistance to anti-PD-1 therapy in lung cancer, we established a novel in vivo model that mimics the clinical evolution of immunotherapy resistance (Fig. 1A). Briefly, LLC cells were subcutaneously implanted into immunocompetent C57BL/6J mice, which were then treated with anti-mouse PD-1 monoclonal antibody (anti-PD-1) or an IgG control. Tumors that progressed under anti-PD-1 treatment in the first cohort were harvested, and small fragments were surgically re-implanted subcutaneously into new, treatment-naive mice. This cycle of treatment and tissue passage was repeated for a total of four rounds to apply selective pressure. Resistance was progressively acquired, as evidenced by the tumor growth curves from each round (Fig. S1A). While anti-PD-1 efficacy was evident in early rounds, the growth curves of the anti-PD-1 and IgG control groups became nearly superimposable by Round 4, confirming the establishment of a stably resistant phenotype. Primary cells were then isolated from these resistant tumor tissues and expanded in vitro to generate a stable cell line-designated LLC-R.

To validate the resistance model, LLC-R cells were reinoculated into naive C57BL/6J mice, with parental LLC cells serving as a treatment-sensitive control. Both groups received either anti-PD-1 antibody or control IgG. As shown in Fig. 1B, anti-PD-1 treatment significantly suppressed tumor growth in mice bearing parental LLC tumors compared to the IgG control. In stark contrast, LLC-R tumors failed to respond to anti-mPD-1 therapy, exhibiting growth kinetics nearly identical to those of the IgG-treated group. These results collectively demonstrate the successful establishment of a robust in vivo resistance model and the derivation of a stably resistant LLC-R cell line.

### KSR2 is significantly upregulated in the anti-PD-1-resistant model

To elucidate the molecular mechanisms underlying resistance to anti-PD-1 therapy, we performed transcriptome sequencing on LLC parental and LLC-R tumor tissues. Our data reveal that the acquired resistance is not primarily driven by direct mutations in tumor cells, but rather by a profound reprogramming of the TME.

Pathway enrichment analysis of DEGs further delineated a remodeled TME characterized by extensive extracellular matrix remodeling and fibrosis, which likely creates a physical barrier impairing T cell infiltration into the tumor core; potent chemokine signaling facilitating the recruitment of inhibitory immune cells; and prominent neutrophil activation and degranulation (Fig. 1E, F, Fig. S1D). These alterations collectively establish both physical and chemical barriers that impede anti-tumor immune responses.

The upregulation of key genes potentially directly involved in immune evasion (Fig. 1C). Notably, we identified a significant upregulation of proprotein convertase subtilisin/kexin type 9 (PCSK9) in resistant tumors. Beyond its established role in cholesterol metabolism [15], PCSK9 can degrade MHC-I on tumor and antigen-presenting cells. Given that MHC-I is essential for T cell recognition, PCSK9 upregulation may enable tumor cells to evade immune surveillance, suggesting a direct mechanism of immune evasion [16].

Concurrently, we focused on KSR2, a critical scaffold protein in the MAPK signaling pathway involved in cell proliferation, metabolism, and survival [17]. Our transcriptomic data confirmed that KSR2 was markedly upregulated in resistant tumors. We hypothesize that KSR2 upregulation may confer resistance by enhancing tumor cell survival or altering metabolic adaptation under therapeutic pressure.

Guided by the sequencing results, we validated the expression of key differentially expressed genes using RT-qPCR. Consistent with our transcriptomic data, *Sorcs2* and *Ksr2* mRNA levels were significantly elevated in LLC-R cells compared to parental LLC cells, whereas *Ubqln2* was downregulated (Fig. 1D). Subsequent Western blot analysis further confirmed the high protein expression of KSR2 in LLC-R cells (Fig. 1G, H).

### *High KSR2 expression associated with poor clinical outcomes and reduced CD8*^+^*T cell infiltration*

**Fig. 2:**
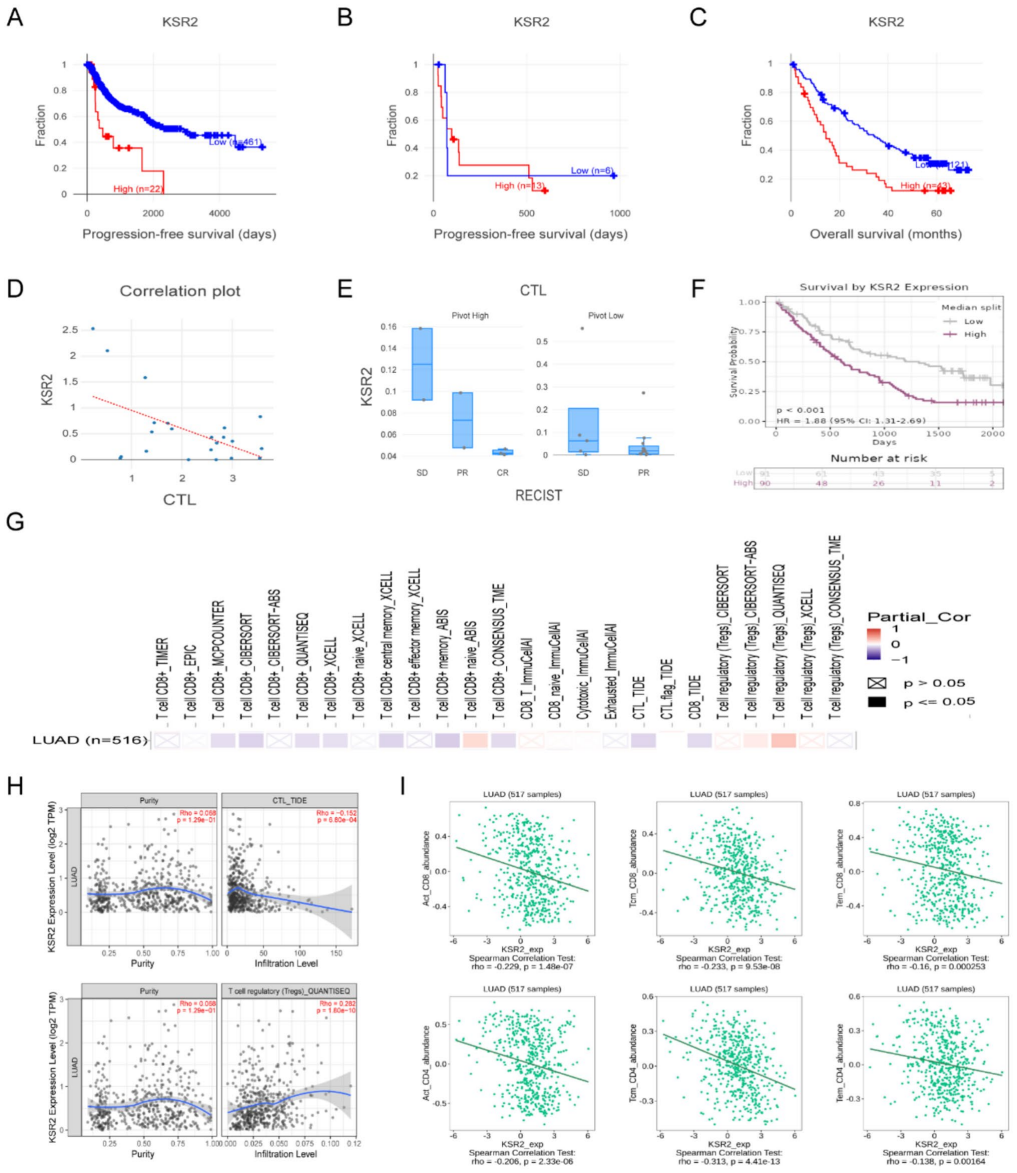
High KSR2 expression correlates with poor clinical outcomes and suppressed T cell infiltration. **A** High *KSR2* expression correlates with shorter PFS in TCGA-LUSC (*z* = 3.74, *p* = 0.000182). **B**, **C** This association is validated in anti-PD-1-treated cohorts of NSCLC (*z* = 2.05, *p* = 0.0401) (**B**) and CCRCC (*z* = 3.41, *p* = 0.000648) (**C**). **D**, **E** In NSCLC, *KSR2* correlates inversely with CTL infiltration (Pearson’s *r* = − 0.53, *p* = 0.012) (**D**) and positively with a T cell dysfunction signature (*t* = − 3.46, *p* = 0.003) (**E**). **F** Kaplan–Meier survival curve for anti-PD-1-treated patients stratified by *KSR2* expression (*z* = 3.98, *p* < 0.0001). **G** A heatmap of Spearman’s correlations between *KSR2* and TIL abundance. **H** In LUAD, *KSR2* shows a negative correlation with CTL but a positive correlation with Treg infiltration (TIMER3.0). **I** In LUAD, *KSR2* is negatively associated with activated and memory T cell subsets (TISIDB)

To evaluate the clinical relevance of KSR2, we leveraged the Cancer Immunology Data Engine (CIDE), an NCI-curated resource integrating large-scale tumor immunotherapy cohorts for analyzing gene expression in relation to treatment response and immune microenvironment features. In the TCGA lung squamous cell carcinoma (LUSC) cohort, higher KSR2 expression was significantly associated with shorter progression-free survival (Cox-PH, z-score = 3.74, *p* = 0.000182; Fig. 2A). To assess its role in the context of immunotherapy, we analyzed an anti-PD-1-treated NSCLC cohort. Consistently, elevated KSR2 expression correlated with poorer progression-free survival, although the cohort size was limited (*n* = 19, z-score = 2.05, *p* = 0.0401) (Fig. 2B). To test generalizability, we analyzed a large, independent cohort of nivolumab-treated clear cell renal cell carcinoma patients (*n* = 164), where high KSR2 expression showed an even more robust correlation with poor survival (z-score = 3.41, *p* = 0.000648; Fig. 2C). These cross-cohort analyses suggest that KSR2 may serve as a potential pan-cancer biomarker associated with resistance to immune checkpoint blockade.

We next investigated the potential immunological mechanisms underlying KSR2-associated poor prognosis. In immunotherapy-treated NSCLC cohorts, KSR2 expression exhibited a significant negative correlation with cytotoxic T lymphocyte infiltration scores (Pearson’s *r* = − 0.528, *p* = 0.0115; Fig. 2D). Concurrently, higher KSR2 expression was strongly associated with an elevated T cell dysfunction signature (t value = − 3.46, *p* = 0.00302; Fig. 2E). These data indicate that KSR2 expression is linked to a “cold” tumor immune microenvironment characterized by reduced cytotoxic T cell presence and increased T cell exhaustion.

We next performed a retrospective meta-analysis using the PRECOG database [18]. Kaplan–Meier analysis demonstrated that high KSR2 expression predicted poorer overall survival in ICI-treated cohorts (HR = 1.88, *P* < 0.01; Fig. 2F), indicating KSR2 as a potential independent adverse prognostic factor in ICI-treated patients.

To further investigate the relationship between KSR2 and immune infiltration, we leveraged TIMER (Tumor Immune Estimation Resource) [19] and TISIDB [20], an integrated repository for tumor–immune system interactions. In lung adenocarcinoma (LUAD), this heatmap depicts the Spearman correlation coefficients (ρ) between KSR2 mRNA expression and the estimated abundances of CD8⁺ T cells and Tregs, as analyzed by the TIMER 3.0 algorithm (Fig. 2G). KSR2 expression was negatively correlated with CD8^+^ T cell abundance but positively correlated with Tregs (Fig. 2H). Pan-cancer correlation analysis across all TCGA tumors is provided in Fig. S1E. Consistent with this, analysis of the TISDB database indicated significant negative Spearman’s correlations between KSR2 expression and multiple activated/memory T cell subsets in LUAD (Fig. 2I). Collectively, these findings indicate that high KSR2 expression is associated with poor clinical outcomes and reduced CD8^+^ T cell infiltration.

### *KSR2 mediates resistance to anti-PD-1 therapy *in vivo

**Fig. 3:**
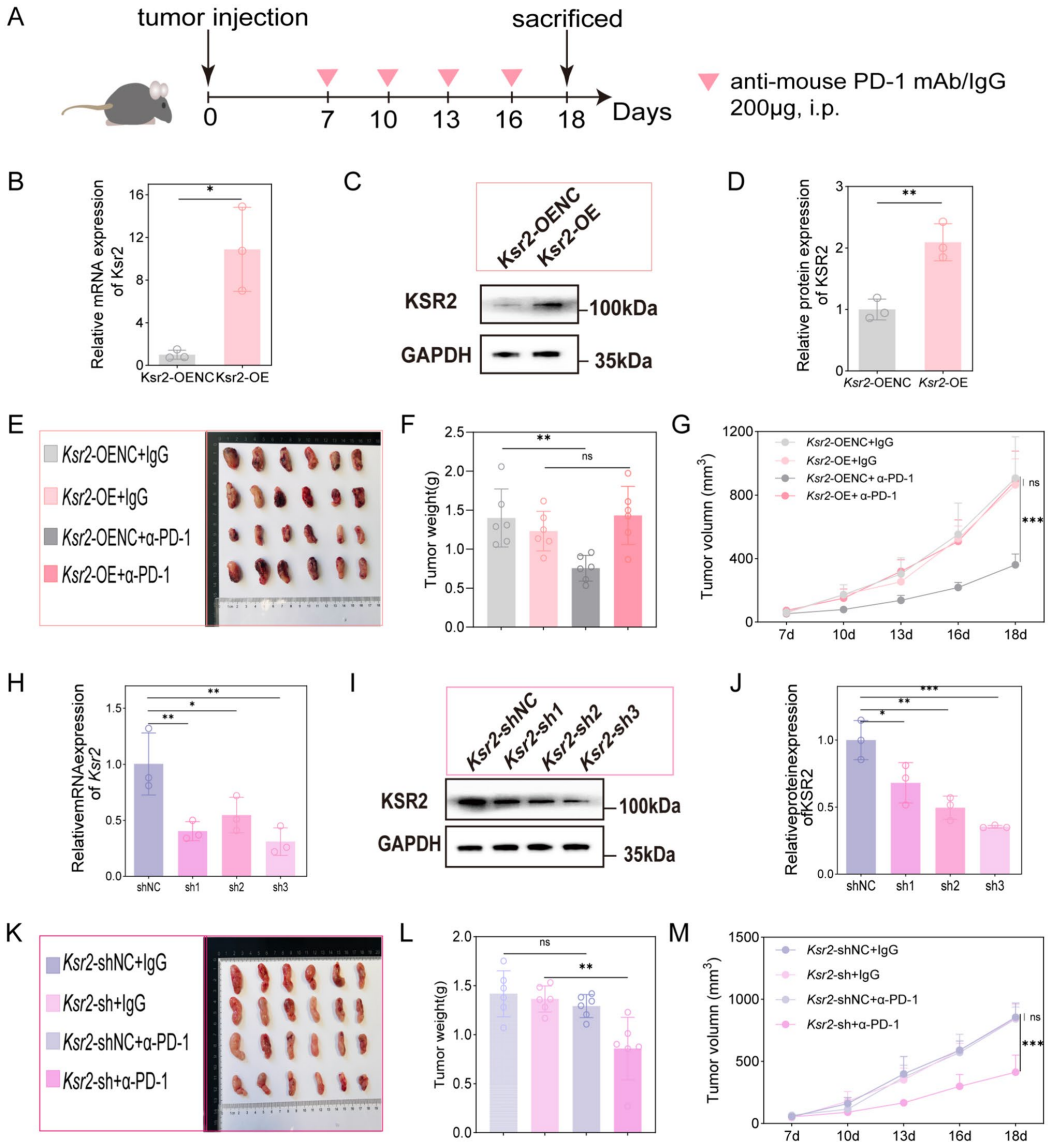
KSR2 is critical for conferring resistance to anti-PD-1 therapy in vivo. **A** Schematic diagram illustrating the in vivo treatment schedule. **B**, **C**, **D** Ksr2 overexpression in LLC stable cell lines validated by RT-qPCR (**B**) and Western blotting (**C**, **D**), mean ± SD (*n* = 3 independent experiments), unpaired two-tailed t test *P < 0.05, **P < 0.01. **E**, **F** Representative tumor images and weights at endpoint. **G** Tumor growth curves in mice bearing control or Ksr2-OE tumors and treated with anti-PD-1 or IgG control (*n* = 6 mice per group). **H**, **I**, **J** *Ksr2* knockdown in LLC-R stable cell lines validated by RT-qPCR (**H**) and Western blotting (**I**, **J**). Mean ± SD (*n* = 3 independent experiments), one-way ANOVA *P < 0.05, **P < 0.01, ***P < 0.001. **K**, **L** Representative tumor images and weights at endpoint. **M** Tumor growth curves in mice bearing control or *Ksr2*-sh tumors and treated with anti-PD-1 or IgG control (*n* = 6 mice per group). Mean ± SD. *P < 0.05, **P < 0.01, ***P < 0.001; NS, not significant, two-way ANOVA (GM); one-way ANOVA (FL)

To investigate whether KSR2 is sufficient to confer resistance to anti-PD-1 therapy in lung cancer, we established stable *Ksr2*-overexpressing (*Ksr2*-OE) and negative control LLC cell lines using a lentiviral vector system. High infection efficiency was confirmed by fluorescence microscopy (Fig. S1B), and RT-qPCR and Western blot analyses demonstrated significant upregulation of *Ksr2* in *Ksr2*-OE cells (Fig. 3B, C, D). These cells were subcutaneously inoculated into the right flank of C57BL/6J mice to generate xenograft models, followed by the administration of either IgG control antibody or anti-mouse PD-1 antibody (Fig. 3A). The results showed that anti-PD-1 treatment significantly suppressed tumor growth in *Ksr2*-OENC mice, but had no notable effect in the *Ksr2*-OE group (Fig. 3E, F, G), with no significant difference in body weight observed between groups (Fig. S1F). These findings indicate that KSR2 overexpression is sufficient to drive resistance to anti-PD-1 therapy.

To further verify the role of KSR2 in mediating anti-PD-1 resistance, we constructed *Ksr2*-knockdown (*Ksr2*-sh) and control stable cell lines in LLC-R cells via lentiviral transduction, employing the most effective shRNA construct (*Ksr2*-sh3) identified in our screening. High infection efficiency was observed by fluorescence imaging (Fig. S1C), and RT-qPCR along with Western blot confirmed pronounced reduction in *Ksr2* expression in *Ksr2*-sh cells (Fig. 3H, I, J). Subsequently, subcutaneous xenograft models were established using these engineered cells, following the same methodology. As illustrated in Fig. 3K, L, M, *Ksr2*-shNC tumors remained insensitive to anti-PD-1 treatment, whereas *Ksr2*-sh tumors exhibited significant growth inhibition, with no marked difference in body weight detected (Fig. S1G). Collectively, our results demonstrate that KSR2 plays a pivotal role in mediating resistance to anti-PD-1 immunotherapy, with its overexpression conferring resistance and its knockdown restoring therapeutic sensitivity.

### KSR2 overexpression associated with an immunosuppressive tumor microenvironment

**Fig. 4:**
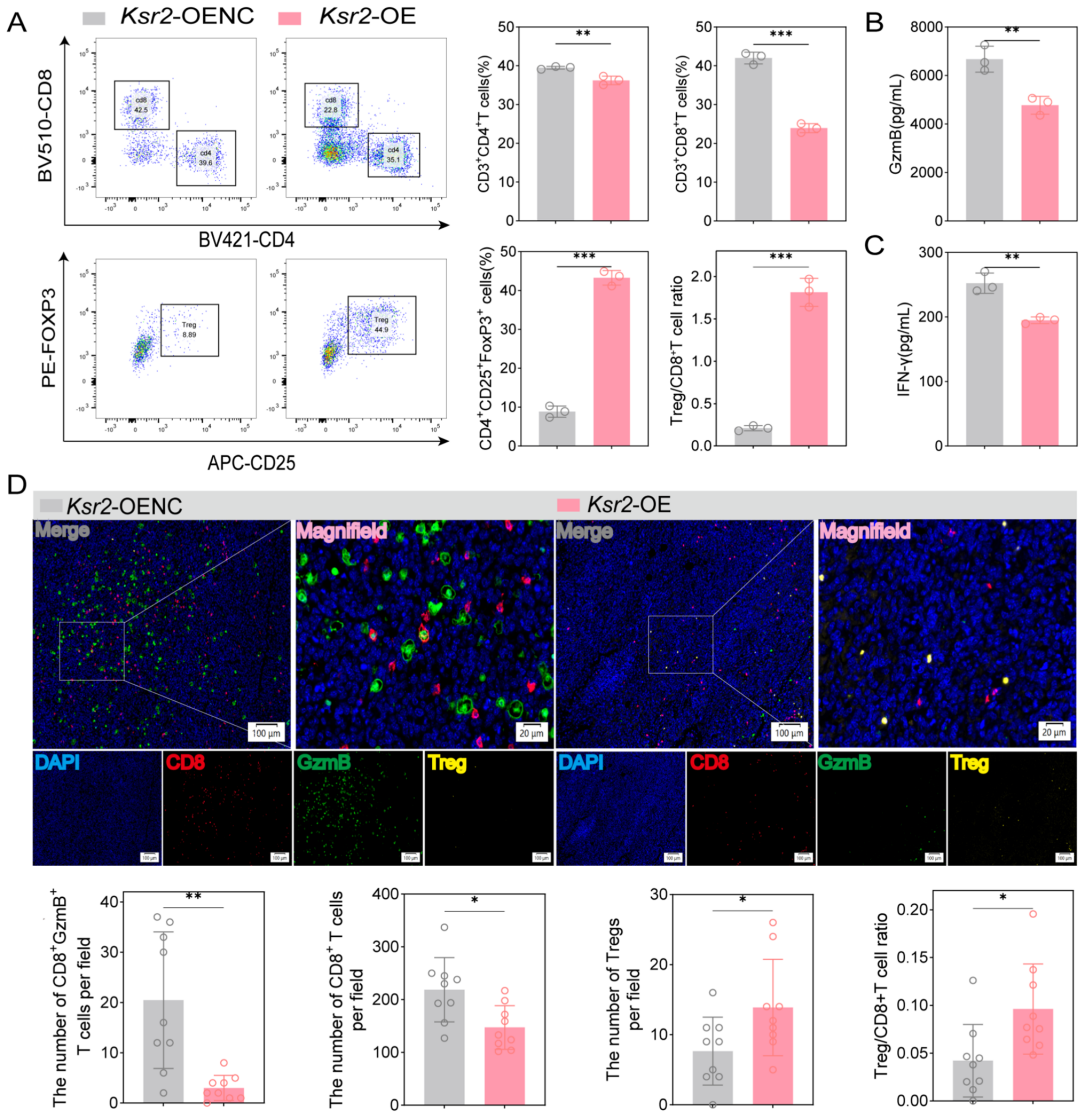
KSR2 overexpression associated with an immunosuppressive tumor microenvironment. **A** Flow cytometry analysis of tumor-infiltrating CD4⁺ T, CD8⁺ T, and regulatory T cells (*n* = 3 mice). **B**, **C** ELISA quantification of GzmB (**B**) and IFN-γ (**C**) levels (*n* = 3 mice). **D** MIF analysis (CD8, red; GzmB, green; Treg, yellow; DAPI, blue) and cell quantification (3 fields/sample; *n* = 3 mice). Mean ± SD. two-tailed unpaired t test or Welch’s t test (*P < 0.05, **P < 0.01, ***P < 0.001)

To elucidate the mechanism by which KSR2 drives resistance to anti-PD-1 therapy, we first sought to characterize its functional impact on the global immune landscape of the TME. We performed flow cytometry analysis on *Ksr2*-OE and control tumors harvested 7 days after initiation of anti-PD-1 therapy. As shown in Fig. 4A, *Ksr2*-OE tumors exhibited a markedly altered immune composition, characterized by a significant reduction in the infiltration of CD4⁺ and CD8⁺ T cells and while markedly increasing the proportion of immunosuppressive Tregs. Consequently, the Treg/CD8⁺ T cell ratio increased significantly and exceeded 1 in *Ksr2*-OE tumors. This initial finding suggested that KSR2 overexpression skews the TME toward immunosuppression by disrupting the critical balance between effector and suppressor cells.

We next employed multiplex immunofluorescence (MIF) to spatially validate these changes within the tumor architecture. Consistent with the flow cytometry data, MIF revealed significantly diminished infiltration of CD8⁺ T cells and a reduction in granzyme B (GzmB) positivity, including a significant drop in double-positive CD8⁺GZMB⁺ effector cells in *Ksr2*-OE tumors (Fig. 4D), which indicates impaired effector cell recruitment and function. In parallel, we observed enhanced Tregs infiltration (Fig. 4D).

To functionally corroborate this immunosuppressive state, we assessed key markers of T cell activation. ELISA quantification of tumor homogenates confirmed a pronounced reduction in the secretion of both GzmB and IFN-γ at the protein level (Fig. 4B, C). The decrease in GzmB, a core cytolytic effector molecule, directly indicates a deficit in tumor-killing capacity. The suppression of IFN-γ, a master regulator of type 1 immunity, reflects inadequate T cell and NK cell activation and suggests a failure to initiate its pleiotropic anticancer functions, including immune stimulation and anti-angiogenesis.

Collectively, these data demonstrate that KSR2 overexpression is accompanied by characteristic remodeling of the tumor microenvironment. Upregulation of KSR2 correlates with reduced infiltration and impaired functionality of cytotoxic T cells, alongside local accumulation of regulatory T cells and suppression of pro-inflammatory cytokine secretion networks. These findings collectively support a model in which KSR2-mediated changes are associated with an immunosuppressive tumor microenvironment.

### KSR2 remodels the tumor glucose metabolic landscape to drive immunosuppression

To further investigate the mechanism by which KSR2 contributes to an immunosuppressive TME, and based on its established role as a critical regulator in the AMP-activated protein kinase (AMPK) pathway governing energy homeostasis and insulin sensitivity [14, 21], we hypothesized that KSR2 influences immune evasion by reprogramming glucose metabolism. To test this hypothesis, we performed comprehensive glucose metabolomics profiling focusing on organic acids and tricarboxylic acid (TCA) cycle intermediates in anti-PD-1-treated *Ksr2*-OE and control (*Ksr2*-OENC) tumor tissues using the ultra-performance liquid chromatography–mass spectrometry platform for targeted metabolite detection and quantification.

**Fig. 5:**
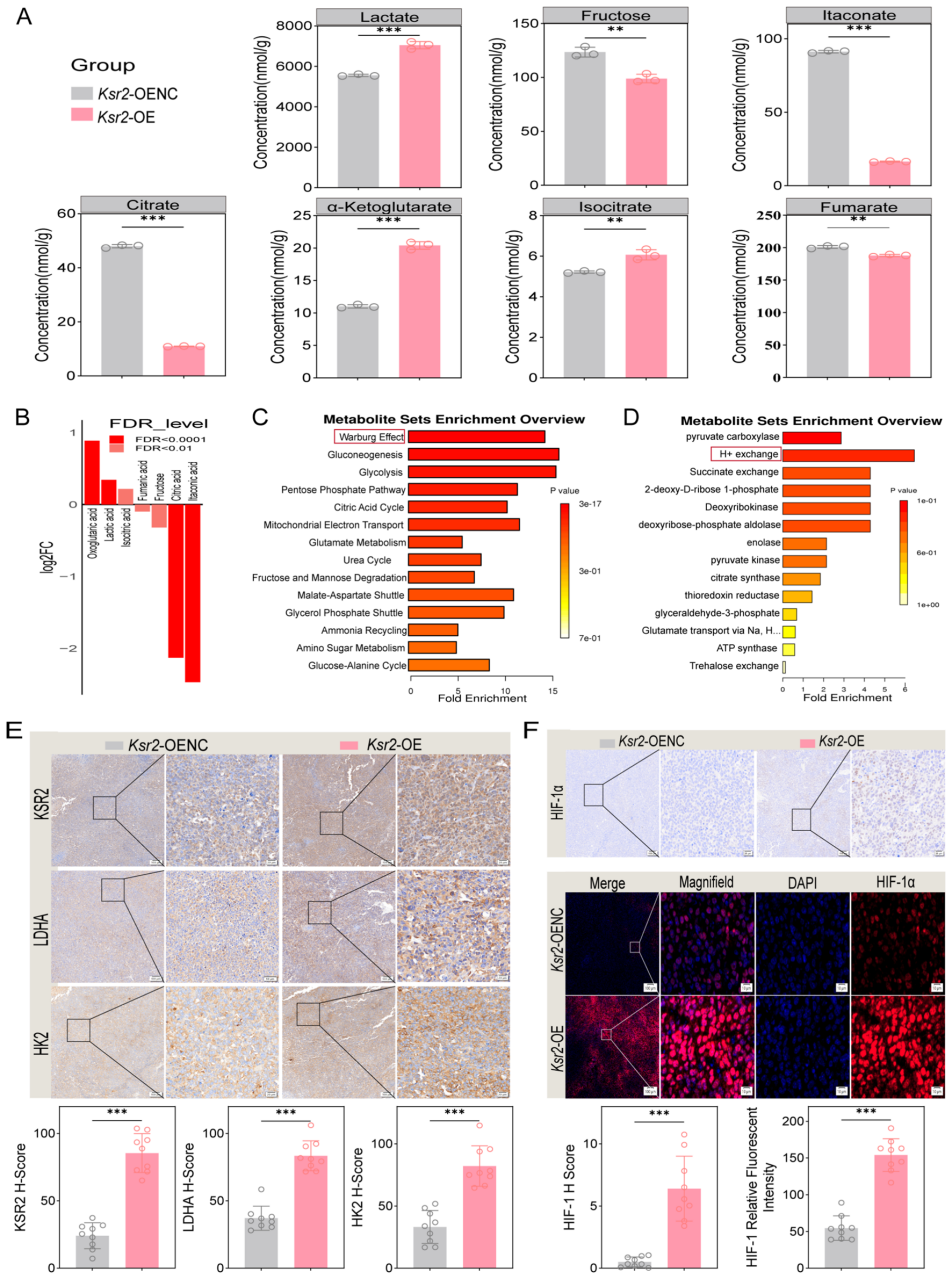
KSR2 overexpression reprograms glucose metabolism in the tumor microenvironment. **A** Abundance of key differentially abundant metabolites between *Ksr2*-OENC and *Ksr2-OE* tumors. Mean ± SD (*n* = 3 mice per group). Unpaired two-tailed t test *P < 0.05, **P < 0.01, ***P < 0.001. **B** Bar plot showing the log2 fold change of significantly altered metabolites. **C**, **D** Pathway enrichment analysis using SMPDB (**C**) and selected predicted metabolite sets (**D**). **E** IHC staining for KSR2, LDHA, and HK2 in *Ksr2*-OE vs. control tumors. 3 fields/sample; *n* = 3 mice. Scale bars: 100μm (main), 20μm (inset). **F** HIF-1α is upregulated and accumulates in the nucleus of *Ksr2*-OE tumors, 3 fields/sample; *n* = 3 mice. As shown by IHC and IF (HIF-1α, red; DAPI, blue). Scale bars: 100μm (main), 20/10μm (insets)

*KSR2 overexpression drives aerobic glycolysis and suggests enhanced glucose uptake.* Metabolomics analysis revealed a significant increase in lactate levels in *Ksr2*-OE tumors (Fig. 5A). As the end-product of glycolysis, elevated lactate accumulation directly indicates enhanced glycolytic flux, reflecting a classic Warburg effect. This finding aligns with the significant enrichment of the “Warburg effect” pathway in metabolic pathway analysis (Fig. 5C). Concurrently, enrichment of the “H^+^ exchange” pathway suggests possible microenvironment acidification resulting from lactate production and efflux (Fig. 5D). Notably, despite active glycolysis, glucose levels in tumor tissues were not decreased but slightly elevated (Fig. S2A). This implies that KSR2 overexpression may substantially enhance tumor cell glucose uptake, sustaining intracellular glucose pools during high glycolysis and potentially leading to competitive glucose deprivation within the TME. Moreover, decreased fructose levels suggest its accelerated utilization as an alternative carbon source to support active biosynthesis.

*KSR2 overexpression remodels the TCA cycle and depletes the immunomodulatory metabolite itaconate.* In *Ksr2*-OE tumors, the TCA cycle underwent profound remodeling, most strikingly characterized by a sharp decline in citrate levels (Fig. 5A). Depletion of citrate, the initiating metabolite of the TCA cycle, typically indicates its export to the cytoplasm for biosynthesis of macromolecules such as fatty acids to meet proliferative demands. Meanwhile, downstream metabolites isocitrate, α-ketoglutarate, and succinate showed accumulation trends (Fig. 5A, B, Fig. S2A). These shifts suggest that under *Ksr2* overexpression, the TCA cycle is redirected from energy production toward supplying precursors for biosynthesis. A key finding was the significant downregulation of itaconate (Fig. 5A, B), an immunomodulatory metabolite, in *Ksr2*-OE tumors. Itaconate is known to inhibit glycolysis. Its depletion may relieve intrinsic suppression of glycolysis, consistent with our observed glycolytic enhancement [22]. Importantly, itaconate has emerged as a potent immunomodulator that can inhibit Treg differentiation and promote pro-inflammatory M1 macrophage polarization, thereby indirectly enhancing CD8⁺ T cell function [23, 24]. Reduction in this immunostimulatory metabolite likely weakens the anti-tumor immune tone in the TME, fostering an immunosuppressive state.

*Biochemical validation of HIF-1α-mediated upregulation of key glycolytic enzymes.* To validate the metabolic rewiring at the protein level, we examined the expression of key glycolytic enzymes. Immunohistochemistry showed significant upregulation of both the glycolytic-initiating enzyme hexokinase 2 (HK2) and the terminal enzyme lactate dehydrogenase A (LDHA) in *Ksr2*-OE tumors (Fig. 5E), providing a direct enzymatic basis for enhanced glucose utilization and lactate production. HIF-1α is a key transcriptional regulator of glycolytic genes including HK2 and LDHA, and we further analyzed hypoxia-inducible factor 1-alpha (HIF-1α) status. Results demonstrated marked stabilization and nuclear accumulation of HIF-1α protein in *Ksr2*-OE tumors (Fig. 5F), indicating activation of its transcriptional activity.

In summary, KSR2 overexpression robustly drives aerobic glycolysis, enhanced glucose uptake, leading to abundant lactate production, which can directly suppress CD8⁺ T cell function and promote Treg generation. Concurrently, KSR2 remodels the TCA cycle toward biosynthesis. Furthermore, depletion of the immunomodulatory metabolite itaconate weakens anti-tumor immune signaling in the TME. We propose that these metabolic alterations shape an immunosuppressive TME rich in lactate, deficient in immunostimulatory metabolites, and dominated by hyperactive anabolism—thereby providing a basis for immune escape under PD-1 blockade therapy.

### KSR2 as a central regulator of metabolic reprogramming

The metabolic profiles observed in vivo represent an integrated output resulting from the interaction between tumor cells and the complex tumor microenvironment. To dissect the intrinsic, cell-autonomous role of KSR2 in regulating tumor cell metabolism, we performed comprehensive energy metabolomics analysis on four isogenic cell lines: LLC-*Ksr2*-OE-NC (LLC), LLC-*Ksr2*-OE, LLC-R-*Ksr2*-shNC (LLC-R), and LLC-R-*Ksr2*-sh. This analysis systematically quantified dynamic changes in metabolites across key pathways, including glycolysis, TCA cycle, pentose phosphate pathway (PPP), and major nitrogen–carbon metabolic hubs.

**Fig. 6:**
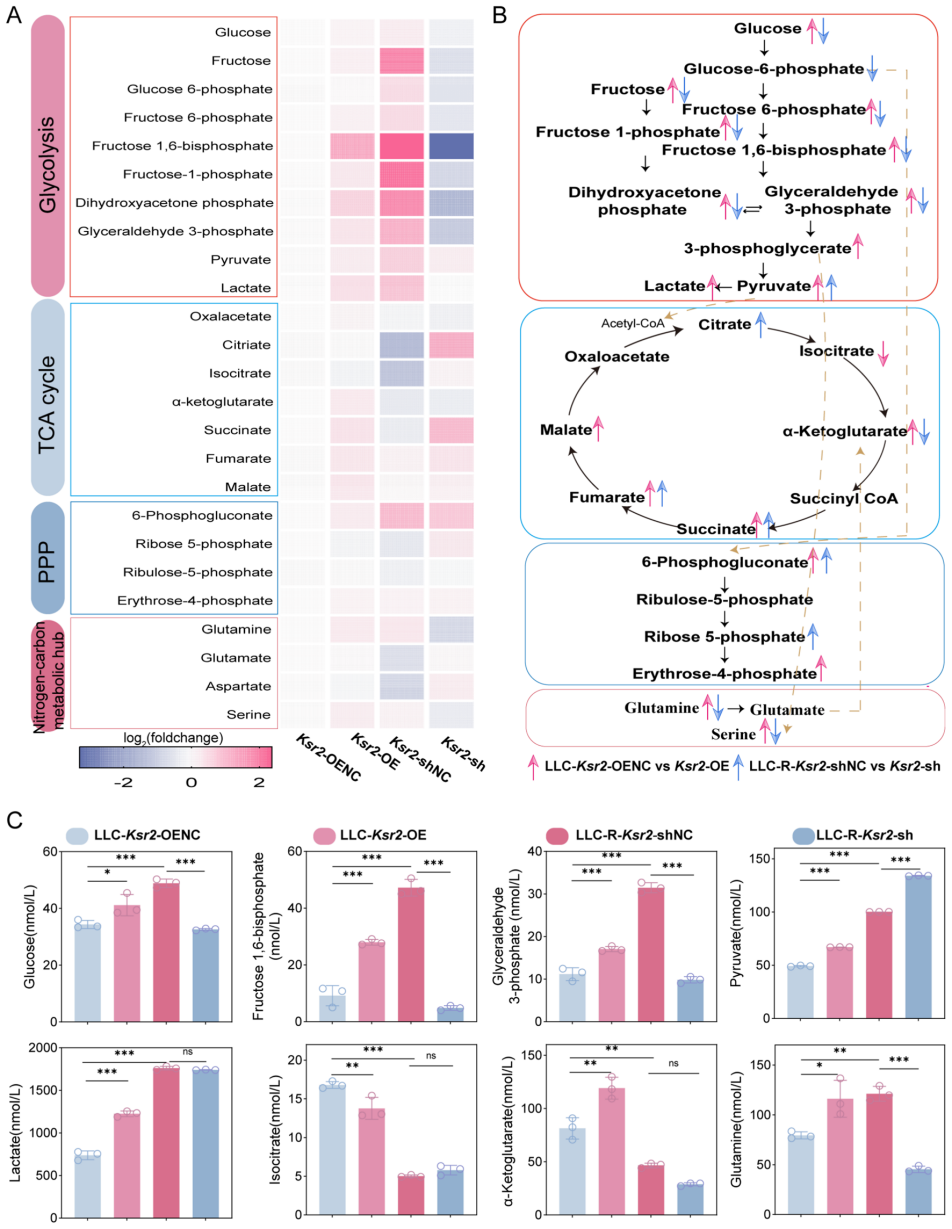
KSR2 acts as a master regulator of metabolic rewiring. **A** Global metabolomics profiles across four isogenic cell lines. Colors represent log2-transformed fold changes relative to the indicated control groups: *Ksr2*-OE and *Ksr2*-shNC (LLC-R) versus *Ksr2*-OENC (LLC); *Ksr2*-sh versus *Ksr2*-shNC. **B** Schematic model depicting flux changes in core metabolic pathways induced by *Ksr2* overexpression or knockdown. **C** Quantitative analysis of key metabolites in isogenic LLC cell lines. Mean ± SD (*n* = 3); one-way ANOVA (*P < 0.05, **P < 0.01, ***P < *0.001,* ns, not significant)

*KSR2 is a central driver of glucose uptake and aerobic glycolysis.* Metabolomics data revealed that KSR2 is a key driver enhancing glucose uptake and glycolytic flux in tumor cells. Overexpression of *Ksr2* in LLC cells significantly increased intracellular glucose levels (Fig. 6C), demonstrating its ability to promote cell-autonomous glucose uptake. This was followed by broad accumulation of glycolytic intermediates: fructose-6-phosphate (F6P), glyceraldehyde 3-phosphate (G3P), 3-phosphoglycerate (3PG), and particularly fructose-1,6-bisphosphate (F1,6BP) (Fig. 6C, Fig. S2C), which increased fourfold–fivefold. Concurrent elevation of downstream pyruvate and the end-product lactate confirmed a comprehensive acceleration of glycolytic flux (Fig. 6C), exhibiting the classic “Warburg effect.” Importantly, KSR2 is required to maintain the hyper-glycolytic phenotype. LLC-R-resistant cells (LLC-R-*Ksr2-*shNC) displayed a similar but more pronounced metabolic phenotype, with glycolytic intermediates and lactate levels significantly higher than those in parental controls and even exceeding *Ksr2*-OE cells, representing a “hyper-activated” state (Fig. 6A). Knockdown of *Ksr2* dramatically reversed this phenotype, intracellular glucose dropped sharply, and key intermediates such as F1, 6BP, and G3P returned to levels comparable to or below those in parental LLC cells (Fig. 6B, C). However, the end-product pyruvate was significantly upregulated, whereas lactate levels showed only a slight, non-significant decrease. In conjunction with the observed changes in glutamine levels, these findings suggest that drug-resistant cells may engage compensatory glutamine catabolism to maintain lactate homeostasis. This indicates that KSR2 is not only an activator of glucose uptake and glycolysis but also a critical node on which resistant tumor cells rely to sustain abnormally high glycolytic flux.

The potent glycolytic capacity driven by KSR2 directly induced functional changes in the microenvironment. *Ksr2*-OE cells secreted large amounts of lactate into the culture medium, significantly reducing extracellular pH, whereas *Ksr2*-knockdown alleviated this acidification (Fig. S1H). This links intracellular metabolic alterations to potential remodeling of the TME, suggesting that *Ksr2*-OE tumor cells may acidify the TME via lactate secretion in vivo, thereby influencing immune cell function.

*KSR2 reprograms the TCA cycle for biosynthetic precursor production.* KSR2 reprograms the TCA cycle, shifting its function from energy production toward supporting biosynthesis. In parental LLC cells, *Ksr2* overexpression remodeled the TCA cycle: Isocitrate levels decreased, whereas downstream intermediates including α-ketoglutarate, succinate, fumarate, and malate accumulated markedly (Fig. 6C, Fig. S2C). This pattern suggests carbon flux diversion at the isocitrate node, likely through enhanced anaplerosis (e.g., glutaminolysis) to maintain flux through the latter part of the cycle, thereby supporting biosynthetic precursors for proliferation rather than complete oxidative energy production. In contrast, KSR2 played a distinct role in resistant LLC-R cells. Control (LLC-R- *Ksr2*-shNC) cells exhibited depletion of the overall TCA metabolite pool, while *Ksr2* knockdown triggered dramatic re-accumulation of metabolites, with citrate levels increasing approximately 2.7-fold (Fig. S2C). This indicates that in the resistant context, KSR2 activity promotes citrate efflux and consumption (e.g., for cytosolic acetyl-CoA and lipid synthesis), forcing the cycle into a “low-accumulation, high-diversion” state. Eliminating KSR2 blocked this diversion, allowing carbon flux to revert and accumulate within the TCA cycle.

*KSR2 coordinates the pentose phosphate pathway and integration of nitrogen–carbon metabolism.* KSR2 further coordinated metabolic networks related to biosynthesis and redox balance. *Ksr2* overexpression enhances the oxidative arm of the pentose phosphate pathway, as indicated by elevated 6-phosphogluconate (Fig. S2C), to meet the increased demand for NADPH—a critical cofactor for anabolic biosynthesis and redox homeostasis. This pathway provides ribose-5-phosphate, the essential backbone for nucleotide synthesis. Further profiling of key hub metabolites revealed how KSR2 coordinates carbon and nitrogen flux. The dissociation between increased glutamine and stable glutamate levels upon *Ksr2*-OE points to accelerated glutaminolysis (Fig. 6C, Fig. S2C), channeling carbon into the TCA cycle as α-ketoglutarate for anaplerosis. Concurrently, the upregulation of serine—a glycolytic branch-point metabolite—bolsters one-carbon unit supply, thereby supporting the escalating needs of nucleotide synthesis, protein production, and antioxidant capacity via glutathione biosynthesis. Conversely, *Ksr2* knockdown reduced both glutamine and serine levels, confirming the essential role of KSR2 in sustaining these anabolic fluxes. KSR2 integrates the PPP with the glutamine–serine metabolic axis, thereby diverting carbon and nitrogen fluxes into biosynthetic pathways. This rewiring ensures a sustained supply of macromolecular building blocks.

This study establishes, at the cell-autonomous level, that KSR2 acts as a central metabolic coordinator that executes a comprehensive reprogramming strategy; it enhances glucose uptake, drives efficient glycolysis and lactate production, remodels the TCA cycle to support biosynthesis, and coordinates the pentose phosphate pathway with carbon/nitrogen metabolism to fuel macromolecule synthesis. The ultimate outcome of this network is a lactate-acidified microenvironment enriched with biosynthetic precursors, which may contribute to tumor proliferation and foster an immunosuppressive TME.

### KSR2 knockdown reverses metabolic reprogramming

To investigate the role of KSR2 in resistance to anti-PD-1 therapy, we systematically analyzed the impact of *Ksr2* knockdown on the metabolic and immune microenvironment in resistant tumor models. Metabolomics data revealed that *Ksr2* knockdown significantly reversed the tumor metabolic phenotype, shifting it from a state characterized by high dependence on glycolysis and a broken TCA cycle toward enhanced oxidative metabolism and accumulation of immunostimulatory metabolites. This reprogramming was consistent with changes in the expression of key metabolic enzymes.

**Fig. 7:**
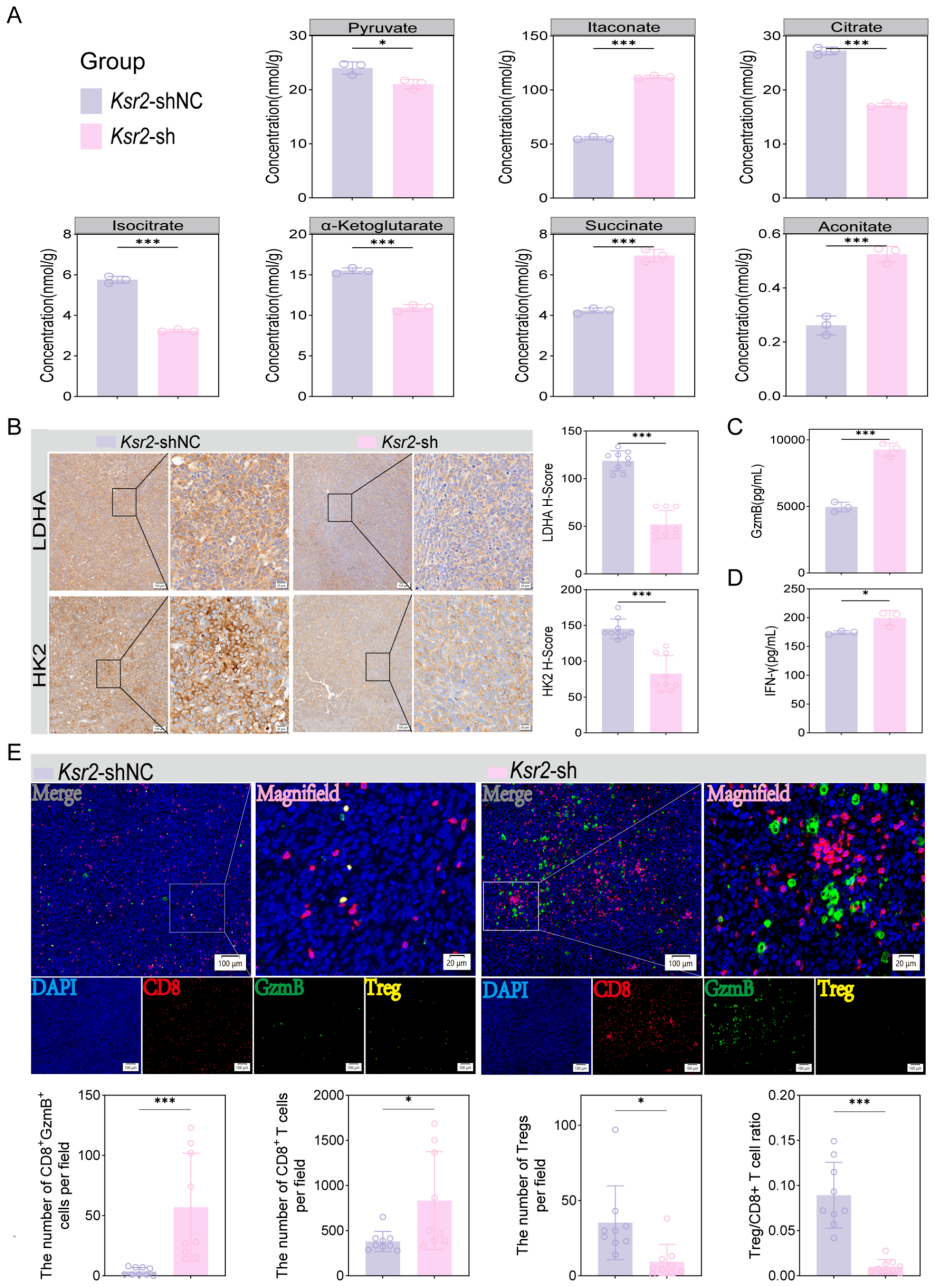
KSR2 knockdown reverses metabolic reprogramming and reinstates anti-tumor immunity. **A** Differential metabolite abundance in control versus Ksr2-knockdown tumors (*n* = 3 mice). **B** IHC analysis of LDHA and HK2 expression. 3 fields/sample; *n* = 3 mice. Scale bars, 100μm, 20*μ*m. **C**, **D** GzmB (**C**) and IFN-γ (**D**) levels in tumors by ELISA (*n* = 3). **E** MIF analysis and cell quantification (3 fields/sample; *n* = 3 mice). Mean ± SD. Two-tailed unpaired t test or Welch’s t test (*P < 0.05, **P < 0.01, ***P < 0.001)

*Ksr2 knockdown reverses the glycolytic phenotype and activates immunostimulatory metabolic pathways.* Targeted metabolomics analysis of tumor tissues showed that glycolysis was significantly suppressed in *Ksr2*-sh tumors, as evidenced by reduced levels of lactate and pyruvate (Fig. 7A, Fig. S2B). Consistent with the in vitro metabolomics results, lactate was not significantly decreased, indicating that glutamine compensation partially exists in vivo. Concurrently, the level of itaconate was markedly increased (Fig. 7A). These changes indicate that the high glycolytic activity of tumor cells was restrained, while immunomodulatory metabolic pathways were activated. Furthermore, glucose levels in tumor tissues were elevated (Fig. S2B), whereas the glucose uptake capacity of tumor cells themselves was weakened, suggesting increased glucose availability for immune cells in the microenvironment. TCA cycle intermediates also exhibited characteristic alterations: citrate, isocitrate, and α-ketoglutarate levels decreased, whereas succinate accumulated, cis- and trans-aconitate and itaconate increased (Fig. 7A), indicating attenuated anaplerotic flux and activation of the Irg1–itaconate axis. This metabolic remodeling was corroborated at the protein level, *Ksr2* knockdown downregulated the expression of HK2 and LDHA (Fig. 7B), and overexpression produced the opposite effect, confirming that KSR2 acts upstream of glycolytic regulation.

### Metabolic remodeling drives the tumor microenvironment toward an immunologically active state

**Fig. 8:**
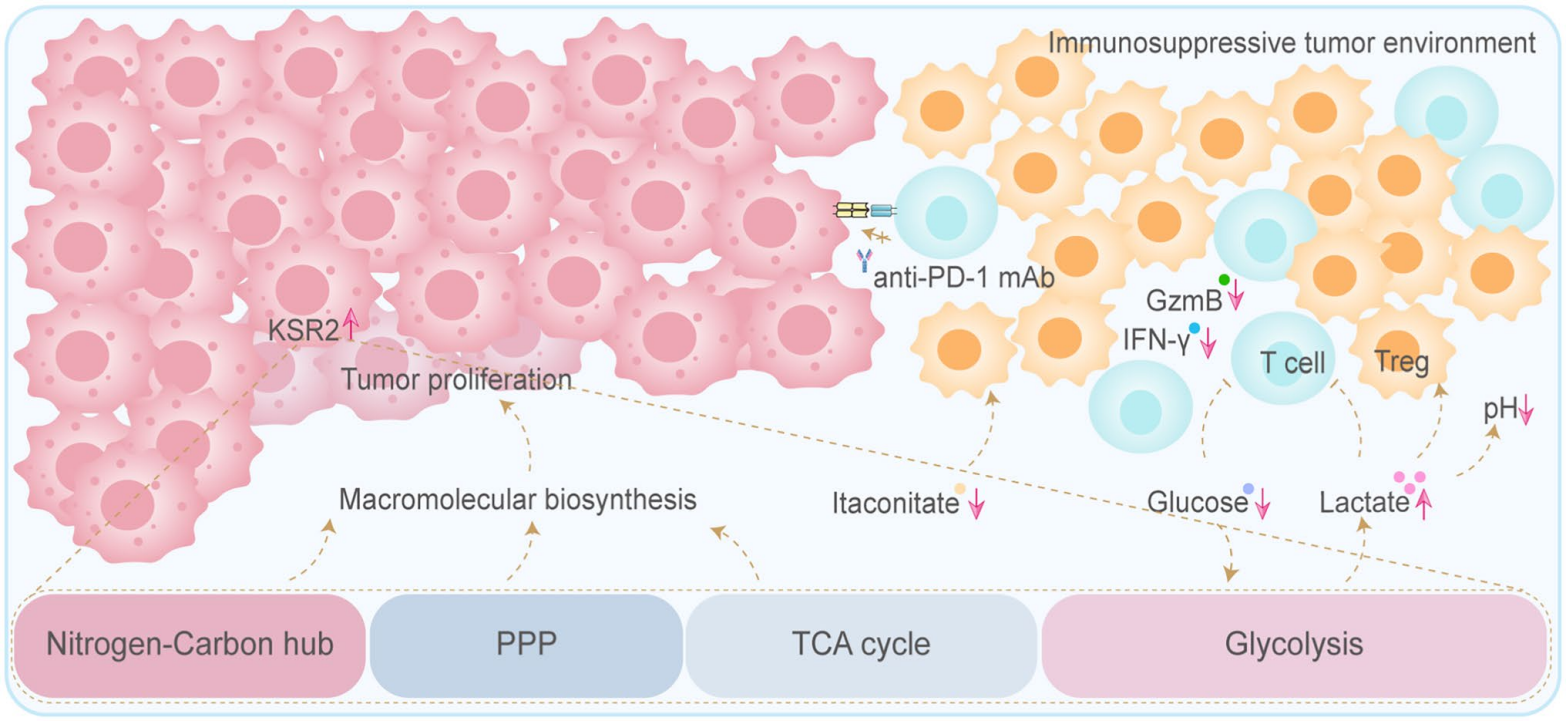
KSR2 overexpression reprograms tumor glucose metabolism to drive anti-PD-1 resistance

Based on our data, we propose that KSR2 remodels the tumor immune microenvironment through the following metabolic mechanisms (Fig. 8).

*Enhancing glucose competition and limiting energy supply for immune cells.* Cellular experiments demonstrated that KSR2 is a central driver of glucose uptake. *Ksr2* overexpression significantly increased intracellular glucose levels in tumor cells, whereas knockdown reduced them, indicating that KSR2 augments autonomous glucose uptake by tumor cells. In tumor tissues, *Ksr2* overexpression maintained tissue glucose levels even under highly active glycolysis, suggesting that tumor cells may monopolize glucose resources in the microenvironment. Conversely, *Ksr2* knockdown led to attenuated glycolysis and a rise in tissue glucose levels. These findings indicate that KSR2 confers a strong competitive advantage for glucose, potentially depleting glucose in the TME and thereby restricting the proliferation and function of CD8⁺ T cells that depend on glucose for energy.

*Promoting lactate accumulation and acidifying and suppressing the immune microenvironment.* Both in vitro culture systems and tumor tissues, *Ksr2* overexpression caused a sharp increase in lactate levels. High lactate concentrations not only directly acidify the TME and inhibit the cytotoxic function and infiltration of CD8⁺ T cells, but have also been widely reported to promote the generation and activity of immunosuppressive Tregs [25, 26]. Thus, KSR2-driven enhancement of glycolytic flux disrupts immune balance through lactate overproduction, simultaneously suppressing effector immunity and enhancing inhibitory immunity.

*Depleting the immunostimulatory metabolite itaconate.* Tissue metabolomics analysis revealed a key alteration that *Ksr2* overexpression led to a significant decrease in itaconate levels in tumors, whereas its knockdown elevated itaconate. Itaconate can indirectly enhance CD8⁺ T cell function by inhibiting Treg differentiation and promoting pro-inflammatory M1-like macrophage polarization. Therefore, KSR2-mediated depletion of itaconate essentially weakens intrinsic immune-activating signals within the TME, further aggravating the immunosuppressive state.

To validate the actual impact of these metabolic mechanisms on the immune microenvironment, we further analyzed the tumor immune phenotype. Multiplex immunofluorescence showed that *Ksr2* knockdown significantly increased CD8⁺ T cell infiltration in tumors and upregulated the activation marker GZMB, including a significant increase in double-positive CD8⁺GZMB⁺ effector cells, while reduced the proportion of Tregs (Fig. 7E). Enzyme-linked immunosorbent assays further confirmed elevated protein levels of GzmB (Fig. 7C) and IFN-γ (Fig. 7D) in the TME. Together, these results demonstrate that targeting KSR2 can reverse the immunosuppressive metabolic environment, converting “cold” tumors into an immunologically “hot” microenvironment, thereby restoring sensitivity to anti-PD-1 therapy.

In summary, this study reveals that in anti-PD-1-resistant models, *Ksr2* knockdown is correlated with remodeling of the tumor immune microenvironment through metabolic reprogramming, including suppression of glycolysis and accumulation of itaconate. These metabolic changes are associated with enhanced CD8⁺ T cell function and reduced Treg activity, contributing to the reversal of immunotherapy resistance. Notably, while lactate levels were not substantially reduced, restoration of glucose availability within the microenvironment appeared to improve T cell effector function. Although direct co-culture experiments are needed to establish causality, these findings identify KSR2 as a putative regulator of metabolism–immune cross talk and raise the possibility that targeting KSR2 may represent a promising combinatorial therapeutic strategy.

## Discussion

Immunotherapy targeting the PD-1/PD-L1 axis has become a cornerstone of treatment for advanced NSCLC, yet the emergence of resistance substantially limits its clinical benefit. This study suggests that KSR2 may serve as a metabolic checkpoint involved in resistance to anti-PD-1 immunotherapy, potentially linking cancer cell metabolic reprogramming to an immunosuppressive tumor microenvironment.

To systematically investigate the role of KSR2 in acquired resistance, an anti-PD-1-resistant lung cancer model was established using LLC cells in immunocompetent C57BL/6J mice. *Ksr2*-overexpressing and knockdown cell lines were generated via lentiviral transduction. In vivo functional studies demonstrated that *Ksr2* overexpression was sufficient to confer resistance to PD-1 blockade, while *Ksr2* knockdown in resistant cells resensitized tumors to treatment. Gain- and loss-of-function experiments confirmed that KSR2 upregulation is not merely a correlative feature but a pivotal driver of resistance. Integrated metabolomics profiling of tumor cells and their surrounding microenvironment revealed KSR2 as a key regulator of glucose metabolism in cancer cells. This KSR2-driven metabolic reprogramming may contribute to tumor cell proliferation and could potentially influence an immunosuppressive microenvironment, possibly through intensified glucose competition, lactate accumulation, acidosis, and reduced levels of the immunostimulatory metabolite itaconate. Flow cytometry and immunofluorescence analyses further revealed that KSR2-mediated metabolic alterations are associated with a suppressive immune landscape, characterized by reduced infiltration of CD8^+^ T cells, diminished granzyme B expression, and an enrichment of regulatory T cells. These findings suggest a potential link between KSR2-mediated metabolic reprogramming and the formation of an immunosuppressive microenvironment, which contributes to the subsequent development of anti-PD-1 resistance.

These observations align closely with the growing consensus that metabolic reprogramming is a central pillar of immune evasion. While KSR2 has been implicated in metabolic control as a scaffold protein, its specific role in orchestrating the multifaceted metabolic programs that underpin immune escape in the context of checkpoint blockade has not been explored. Our correlative findings suggest a possible link among KSR2, metabolic reprogramming, and the immune microenvironment. We propose that KSR2 may reprogram glucose metabolism to promote an immunosuppressive milieu, which could in turn impair anti-tumor immunity.

The findings presented here suggest potential translational implications. The identified axis offers a conceptual framework for understanding the metabolic regulation of immunotherapy response. KSR2 may represent a candidate predictive biomarker for anti-PD-1 efficacy and a possible therapeutic target. Targeting KSR2 function might help restore metabolic homeostasis within the tumor microenvironment and reinvigorate anti-tumor immunity, representing a novel strategy to overcome resistance to immunotherapy.

Despite these advances, several limitations warrant consideration in future studies. First, the current findings are derived from subcutaneous syngeneic tumor models using only female mice. While this model provides a complete immune system and is an indispensable tool for immunotherapy research, it does not fully recapitulate the complex tumor–stroma interactions within the native lung microenvironment. Future work should incorporate orthotopic tumor models that preserve tissue architecture and include both sexes to validate the generalizability of these conclusions and to assess potential sex dimorphism in KSR2-mediated metabolic reprogramming. Second, although a strong correlation was observed between KSR2 expression in tumor cells and the extent of CD8^+^ T cell and regulatory T cell infiltration, causal evidence that tumor cell-intrinsic KSR2 directly regulates the function of specific immune subsets remains incomplete. Co-culture experiments using KSR2-modified tumor cells with T cells or regulatory T cells would help clarify whether the observed immune remodeling is a direct consequence of cancer cell-autonomous metabolic effects or is indirectly mediated through alterations in the microenvironmental milieu. Third, while the data reveal a link between KSR2-driven metabolic reprogramming and immunosuppression, direct mechanistic evidence requires in vivo rescue experiments. For instance, neutralizing lactate, supplementing itaconate, or buffering acidosis in *Ksr2*-overexpressing tumors would help establish whether these metabolites are causally necessary for mediating immune evasion and anti-PD-1 resistance. Finally, we are currently assembling a cohort of patients with non-small cell lung cancer receiving anti-PD-1 therapy to systematically evaluate associations between KSR2 expression, tumor microenvironment metabolic features, immune infiltration patterns, and clinical outcomes. This effort aims to translate these mechanistic findings into potential biomarkers and combination therapeutic strategies. Addressing these limitations in future research to definitively establish the causal mechanisms linking KSR2-driven metabolic reprogramming and immunosuppression will be crucial for refining this conceptual framework and advancing its clinical translation.

In summary, based on integrating transcriptomic, metabolomics, and functional in vivo analyses, this study suggests that KSR2 drives resistance to anti-PD-1 therapy in lung cancer. The work further indicates that KSR2 is a key regulator of glucose metabolism, where its overexpression enhances glucose uptake and glycolysis while remodeling the tricarboxylic acid cycle. This KSR2-mediated metabolic reprogramming correlates with an immunosuppressive tumor microenvironment. Notably, direct causal evidence, particularly from T cell or tumor cell co-culture experiments, remains to be established. Nevertheless, these findings position KSR2 as a candidate metabolic node worthy of further investigation. Targeted knockdown of *Ksr2* restored treatment sensitivity in resistant models, suggesting that modulating KSR2 function may represent a potential therapeutic strategy to overcome immunotherapy resistance, pending validation in future mechanistic studies.

## Supplementary Information

Below is the link to the electronic supplementary material.

Supplementary file1 (PDF 884 kb)

## Data Availability

The datasets generated during and analysed during the current study are available from the corresponding author on reasonable request.

## References

[1] Brody H (2020) Lung cancer. Nature 587(7834):S7. 10.1038/d41586-020-03152-0 33208969 10.1038/d41586-020-03152-0

[2] Siegel RL, Kratzer TB, Giaquinto AN, Sung H, Jemal A (2025) Cancer statistics, 2025. CA Cancer J Clin 75(1):10–45. 10.3322/caac.21871 39817679 10.3322/caac.21871 PMC11745215

[3] Sung H, Ferlay J, Siegel RL et al (2021) Global cancer statistics 2020: GLOBOCAN estimates of incidence and mortality worldwide for 36 cancers in 185 countries. CA Cancer J Clin 71(3):209–249. 10.3322/caac.21660 33538338 10.3322/caac.21660

[4] Reck M, Remon JHellmann MD (2022) First-line immunotherapy for non-small-cell lung cancer. J Clin Oncol 40(6):586–597. 10.1200/jco.21.01497 34985920 10.1200/JCO.21.01497

[5] Lahiri A, Maji A, Potdar PD et al (2023) Lung cancer immunotherapy: progress, pitfalls, and promises. Mol Cancer 22(1):40. 10.1186/s12943-023-01740-y 36810079 10.1186/s12943-023-01740-y PMC9942077

[6] Pang K, Shi ZD, Wei LY et al (2023) Research progress of therapeutic effects and drug resistance of immunotherapy based on PD-1/PD-L1 blockade. Drug Resist Updat 66:100907. 10.1016/j.drup.2022.100907 36527888 10.1016/j.drup.2022.100907

[7] Vesely MD, Zhang TChen L (2022) Resistance mechanisms to anti-PD cancer immunotherapy. Annu Rev Immunol 40:45–74. 10.1146/annurev-immunol-070621-030155 35471840 10.1146/annurev-immunol-070621-030155

[8] Hu J, Zhang L, Xia H et al (2023) Tumor microenvironment remodeling after neoadjuvant immunotherapy in non-small cell lung cancer revealed by single-cell RNA sequencing. Genome Med 15(1):14. 10.1186/s13073-023-01164-9 36869384 10.1186/s13073-023-01164-9 PMC9985263

[9] Liu S, Zhang X, Wang W et al (2024) Metabolic reprogramming and therapeutic resistance in primary and metastatic breast cancer. Mol Cancer 23(1):261. 10.1186/s12943-024-02165-x 39574178 10.1186/s12943-024-02165-x PMC11580516

[10] Zhang C, Wang H, Liu Q et al (2023) LncRNA CCAT1 facilitates the progression of gastric cancer via PTBP1-mediated glycolysis enhancement. J Exp Clin Cancer Res 42(1):246. 10.1186/s13046-023-02827-6 37740243 10.1186/s13046-023-02827-6 PMC10517515

[11] Wang J, Jia W, Zhou X et al (2024) CBX4 suppresses CD8(+) T cell antitumor immunity by reprogramming glycolytic metabolism. Theranostics 14(10):3793–3809. 10.7150/thno.95748 38994031 10.7150/thno.95748 PMC11234269

[12] Kumagai S, Koyama S, Itahashi K et al (2022) Lactic acid promotes PD-1 expression in regulatory T cells in highly glycolytic tumor microenvironments. Cancer Cell 40(2):201-218.e9. 10.1016/j.ccell.2022.01.001 35090594 10.1016/j.ccell.2022.01.001

[13] Liu Y, Liang G, Xu H et al (2021) Tumors exploit FTO-mediated regulation of glycolytic metabolism to evade immune surveillance. Cell Metab 33(6):1221-1233.e11. 10.1016/j.cmet.2021.04.001 33910046 10.1016/j.cmet.2021.04.001

[14] Pearce LR, Atanassova N, Banton MC et al (2013) KSR2 mutations are associated with obesity, insulin resistance, and impaired cellular fuel oxidation. Cell 155(4):765–777. 10.1016/j.cell.2013.09.058 24209692 10.1016/j.cell.2013.09.058 PMC3898740

[15] Bao X, Liang Y, Chang H et al (2024) Targeting proprotein convertase subtilisin/kexin type 9 (PCSK9): from bench to bedside. Signal Transduct Target Ther 9(1):13. 10.1038/s41392-023-01690-3 38185721 10.1038/s41392-023-01690-3 PMC10772138

[16] Liu X, Bao X, Hu M et al (2020) Inhibition of PCSK9 potentiates immune checkpoint therapy for cancer. Nature 588(7839):693–698. 10.1038/s41586-020-2911-7 33177715 10.1038/s41586-020-2911-7 PMC7770056

[17] Gao C, Wang SW, Lu JC et al (2022) KSR2-14-3-3ζ complex serves as a biomarker and potential therapeutic target in sorafenib-resistant hepatocellular carcinoma. Biomark Res 10(1):25. 10.1186/s40364-022-00361-9 35468812 10.1186/s40364-022-00361-9 PMC9036720

[18] Gentles AJ, Newman AM, Liu CL et al (2015) The prognostic landscape of genes and infiltrating immune cells across human cancers. Nat Med 21(8):938–945. 10.1038/nm.3909 26193342 10.1038/nm.3909 PMC4852857

[19] Li T, Fu J, Zeng Z et al (2020) TIMER2.0 for analysis of tumor-infiltrating immune cells. Nucleic Acids Res 48(W1):W509-w514. 10.1093/nar/gkaa407 32442275 10.1093/nar/gkaa407 PMC7319575

[20] Ru B, Wong CN, Tong Y et al (2019) TISIDB: an integrated repository portal for tumor-immune system interactions. Bioinformatics 35(20):4200–4202. 10.1093/bioinformatics/btz210 30903160 10.1093/bioinformatics/btz210

[21] Costanzo-Garvey DL, Pfluger PT, Dougherty MK et al (2009) KSR2 is an essential regulator of AMP kinase, energy expenditure, and insulin sensitivity. Cell Metab 10(5):366–378. 10.1016/j.cmet.2009.09.010 19883615 10.1016/j.cmet.2009.09.010 PMC2773684

[22] McGettrick AF, Bourner LA, Dorsey F CO’Neill LAJ (2024) Metabolic messengers: itaconate. Nat Metab 6(9):1661–1667. 10.1038/s42255-024-01092-x 39060560 10.1038/s42255-024-01092-x

[23] Domínguez-Andrés J, Novakovic B, Li Y et al (2019) The itaconate pathway is a central regulatory node linking innate immune tolerance and trained immunity. Cell Metab 29(1):211-220.e5. 10.1016/j.cmet.2018.09.003 30293776 10.1016/j.cmet.2018.09.003

[24] Chen F, Elgaher WAM, Winterhoff M et al (2022) Citraconate inhibits ACOD1 (IRG1) catalysis, reduces interferon responses and oxidative stress, and modulates inflammation and cell metabolism. Nat Metab 4(5):534–546. 10.1038/s42255-022-00577-x 35655026 10.1038/s42255-022-00577-x PMC9170585

[25] Ye L, Jiang YZhang M (2022) Crosstalk between glucose metabolism, lactate production and immune response modulation. Cytokine Growth Factor Rev 68:81–92. 10.1016/j.cytogfr.2022.11.001 36376165 10.1016/j.cytogfr.2022.11.001

[26] Apostolova PPearce EL (2022) Lactic acid and lactate: revisiting the physiological roles in the tumor microenvironment. Trends Immunol 43(12):969–977. 10.1016/j.it.2022.10.005 36319537 10.1016/j.it.2022.10.005 PMC10905416

