# Supplementary material for: KSR2 functions as a metabolic checkpoint for anti-PD-1 resistance by reprogramming glucose metabolism
Source: Cancer Immunol Immunother. 2026 Apr 21;75(5):151. doi: 10.1007/s00262-026-04394-z (PMC13100183; doi:10.1007/s00262-026-04394-z)
Supplement: Supplementary file 1 — Supplementary file1 (PDF 884 kb) [file 262_2026_4394_MOESM1_ESM.pdf]

supplementary figure 1

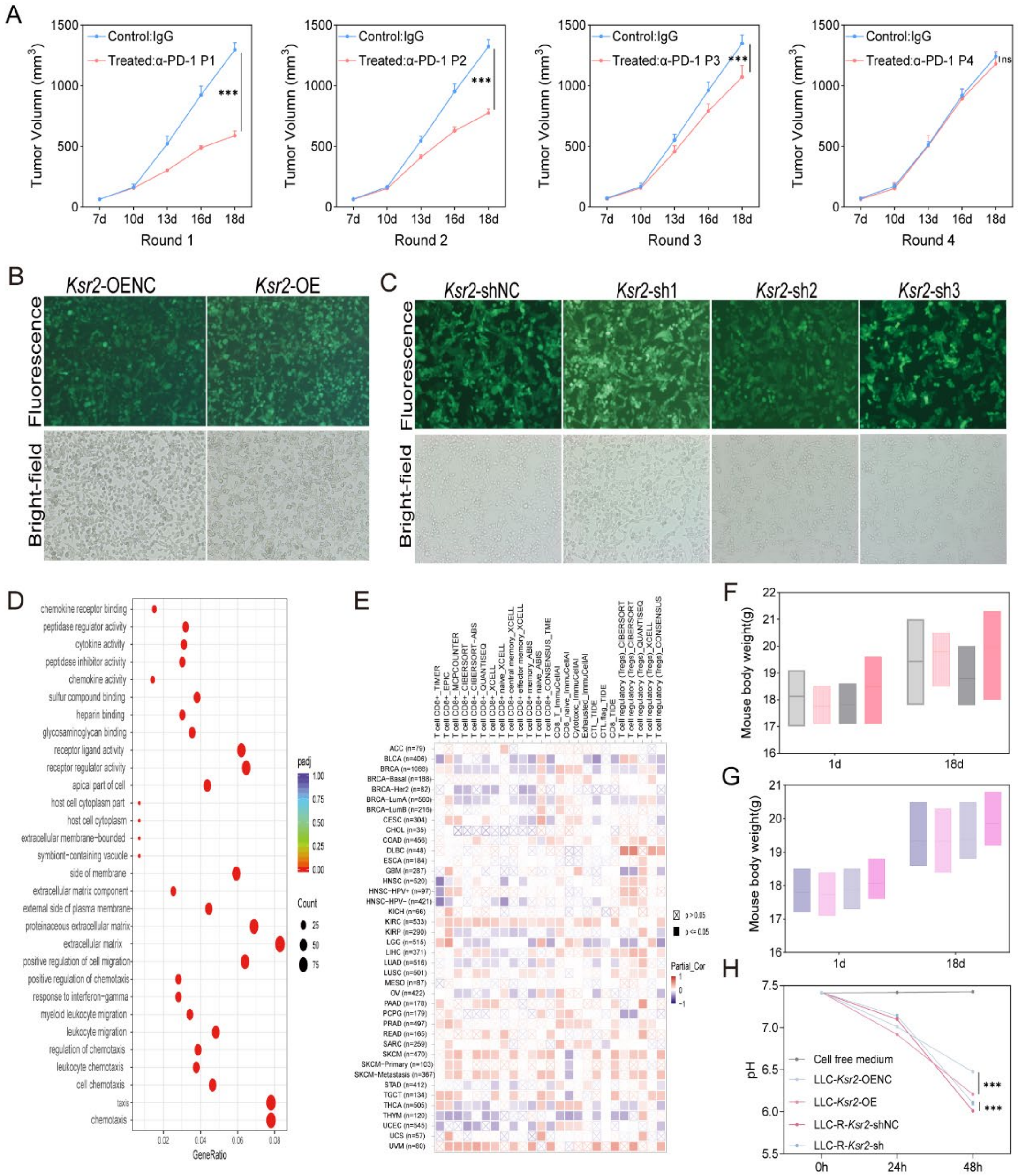

Functional analysis of KSR2 expression in tumor models. (A) The establishment of the anti-PD-1 resistance mouse model. (BC) Representative fluorescence microscopy images of *Ksr2*-manipulated cells. (D) Pathway enrichment analysis (GO) of DEGs. (E) Correlation between KSR2 expression and immune cell infiltration. (FG) Body weight changes of tumor-bearing mice harboring *Ksr2*-OE (F) or *Ksr2*-sh (G) tumors at day 1 and day 18 of treatment (n=6 per group). (H) Dynamic changes in supernatant pH from cultured four isogenic cell lines. mean ± SD (n=3), two-way ANOVA, ***$p < 0.001$.

supplementary figure 2

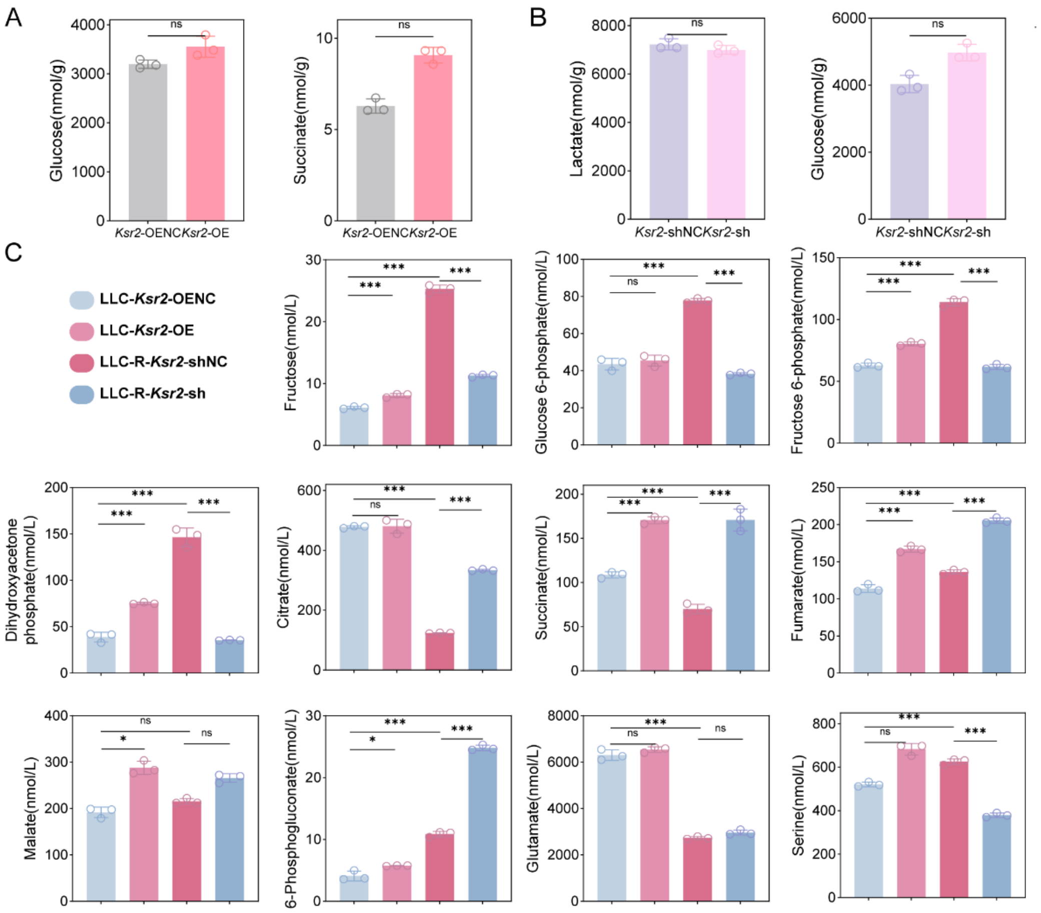

Metabolite analysis of glucose metabolism in tumor tissues and cells. (A) Relative levels of glucose and succinate metabolites in *Ksr2*-OE tumor tissues compared to negative control tissues. mean ± SD (n= 3 mice per group). Unpaired t test; ns, not significant. (B) Relative levels of lactate and glucose metabolites in Ksr2-sh tumor compared to Ksr2-NC tumor. mean ± SD (n= 3 mice per group). Mann-Whitney test; ns, not significant. (C) Analysis of intracellular energetic metabolites in the corresponding isogenic LLC cell lines under basal conditions. mean ± SD (n=3); one-way ANOVA (*P < 0.05, **P < 0.01, ****P* < *0.001,* ns, not significant*).*

supplementary figure 3

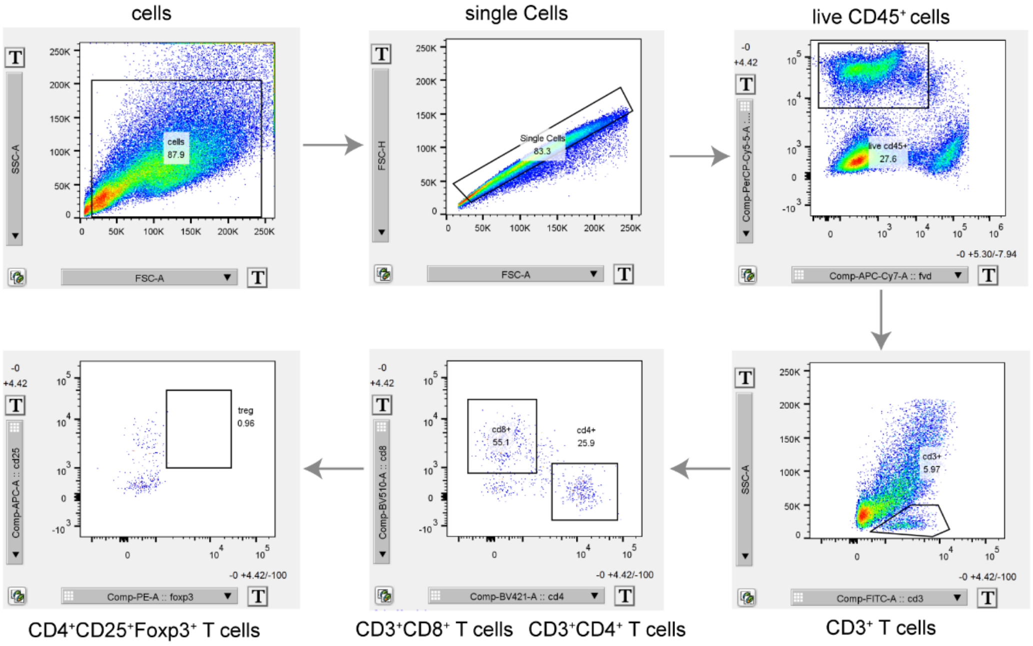

Flow cytometry gating strategy

supplementary table1

Gene	Forward Primer	Reverse Primer
GAPDH	CATCACTGCCACCCAGAAGACTG	ATGCCAGTGAGCTTCCCGTTCAG
Sorcs2	GAGCACCATGTGTTGTACCTGG	CATCCACGAACACTGAGGTGCT
Rhou	TGGTCAGCTACACCACTAACGG	TGCAGTGTCACAGAGCTGGAGT
Ubqln2	ATGAGGCAGACGCTGGAAATCG	CTGTGCGGCATTCAGCATAGGT
Ksr2	GTACTGGATGTCTCAGACGTGC	CTCGGTGGATAATCAGGAGGTG

Primer sequences used in the study for RT-qPCR.

supplementary table 2

Name	Forward Primer	Reverse Primer
Ksr2	GATCTATTTCCGGTGAATTCCGCCACCATGGATGAGGAAAACATGACGAAAAGC	TCCTTGTAGTCCATGGATCCCAGCTCTGCAGACTTCCAGAAATGTCC
Ksr2 sh1	CCGGGCCTGCCCGATATTCAGATCTCTCGAGAGATCTGAATATCGGGCAGGCTTTTTG	AATTCAAAAAGCCTGCCCGATATTCAGATCTCTCGAGAGATCTGAATATCGGGCAGGC
Ksr2 sh2	CCGGGGACAATGAGGACCAGCTTAACTCGAGTTAAGCTGGTCCTCATTGTCCTTTTTG	AATTCAAAAAGGACAATGAGGACCAGCTTAACTCGAGTTAAGCTGGTCCTCATTGTCC
Ksr2 sh3	CCGGGCTTGGCACCATCTGGTATGACTCGAGTCATACCAGATGGTGCCAAGCTTTTTG	AATTCAAAAAGCTTGGCACCATCTGGTATGACTCGAGTCATACCAGATGGTGCCAAGC

Primer sequences used in the study for lentivirus construction.
